# Acute Kidney Injury in Critically-Ill COVID-19 Patients

**DOI:** 10.3390/jcm11072029

**Published:** 2022-04-05

**Authors:** Romain Arrestier, Ségolène Gendreau, David Mokrani, Jean-Philippe Bastard, Soraya Fellahi, François Bagate, Paul Masi, Thomas d’Humières, Keyvan Razazi, Guillaume Carteaux, Nicolas De Prost, Vincent Audard, Armand Mekontso-Dessap

**Affiliations:** 1 Service de Médecine Intensive Réanimation, Assistance Publique-Hôpitaux de Paris (AP-HP), Hôpitaux Universitaires Henri Mondor, 94010 Creteil, France; segolene.gendreau@aphp.fr (S.G.); david.mokrani@gmail.com (D.M.); francois.bagate@php.fr (F.B.); paul.masi@aphp.fr (P.M.); keyvan.razazi@aphp.fr (K.R.); guillaume.carteaux@aphp.fr (G.C.); nicolas.de-prost@aphp.fr (N.D.P.); armand.dessap@aphp.fr (A.M.-D.); 2 GRC CARMAS, Faculté de Médecine de Créteil, Université Paris Est Créteil, 94010 Creteil, France; 3 Département de Biochimie-Pharmacologie-Biologie Moléculaire-Génétique Médicale, Assistance Publique-Hôpitaux de Paris (AP-HP), Hôpitaux Universitaires Henri Mondor, 94010 Creteil, France; jean-philippe.bastard@aphp.fr (J.-P.B.); soraya.fellahi@aphp.fr (S.F.); 4 Institut National de la Santé et de la Recherche Médicale (INSERM) U955, Institut Mondor de Recherche Biomédicale (IMRB), Université Paris Est Créteil, 94010 Creteil, France; vincent.audard@aphp.fr; 5 Centre de Recherche Saint-Antoine, Institut Hospitalo-Universitaire de Cardio-Métabolisme et Nutrition (ICAN), Institut National de la Santé et de la Recherche Médicale (INSERM) UMR S938, Sorbonne Université, 75006 Paris, France; 6 Service de Physiologie Explorations Fonctionnelles, Assistance Publique-Hôpitaux de Paris (AP-HP), Hôpitaux Universitaires Henri Mondor, 94010 Creteil, France; 7 Service de Néphrologie et Transplantation, Centre de Référence Maladie Rare Syndrome Néphrotique Idiopathique, Assistance Publique-Hôpitaux de Paris (AP-HP), Fédération Hospitalo-Universitaire Innovative Therapy for Immune Disorders, Hôpitaux Universitaires Henri Mondor, 94010 Creteil, France

**Keywords:** acute kidney injury, tubular necrosis, COVID, SARS-CoV2, dialysis, proteinuria

## Abstract

Purpose: Acute kidney injury (AKI) is common in patients with COVID-19, however, its mechanism is still controversial, particularly in ICU settings. Urinary proteinuria profile could be a non-invasive tool of interest to scrutinize the pathophysiological process underlying AKI in COVID-19 patients. Material and Methods: We conducted a retrospective study between March 2020 and April 2020. All patients with laboratory-confirmed COVID-19 and without end-stage kidney disease requiring renal replacement therapy before ICU admission were included. Our objectives were to assess the incidence and risk factors for AKI and to describe its clinical and biological characteristics, particularly its urinary protein profile. Results: Seventy patients were included; 87% needed mechanical ventilation and 61% needed vasopressor during their ICU stay; 64.3% of patients developed AKI and half of them needed dialysis. Total and tubular proteinuria on day 1 were higher in patients with AKI, whereas glomerular proteinuria was similar in both groups. The main risk factor for AKI was shock at admission (OR = 5.47 (1.74–17.2), *p* < 0.01). Mortality on day 28 was higher in AKI (23/45, 51.1%) than in no-AKI patients (1/25, 4%), *p* < 0.001. Risk factors for 28-days mortality were AKI with need for renal replacement therapy, non-renal SOFA score and history of congestive heart failure. Conclusions: AKI is common in COVID-19 patients hospitalized in ICU; it seems to be related to tubular lesions rather than glomerular injury and is related to shock at ICU admission.

## 1. Introduction

The main feature of COVID-19 is viral pneumonia with microvascular thrombosis leading to acute respiratory distress syndrome (ARDS) in some patients, of whom 10 to 30% need intensive care admission [1,2]. The pathophysiological process underlying severe COVID-19 is still controversial, but several studies have suggested that angiotensin-converting enzyme 2 (ACE2), which is expressed by many human cells, including kidney cells, may play a prominent role in intracellular invasion [3,4,5]. Kidney involvement, presented by a broad spectrum of renal manifestations, seems to be common in SARS-CoV2 infected patients and its reported incidence largely varies between studies. First studies reported variable incidence of acute kidney injury (AKI) from 0 to 14.7%, and in intensive care unit (ICU) settings, it reaches 8.3 to 28.8% [6]; these data primarily come from Chinese population-based studies [7,8]. More recent studies have documented a higher AKI incidence amounting to 35% in hospitalized patients in America [9] and up to 50–80% in ICU [10,11,12]. However, the relative role of glomerular versus tubular injury is still debated. In addition, severe COVID-19-related shock and ARDS may induce complex hemodynamic compromise altering heart contractility and loading conditions, which may affect kidney function.

Kidney biopsy is the gold standard to investigate mechanisms involved in AKI occurrence, but due to the hemodynamic instability and hemostasis alteration, it is rarely performed in ICU settings. Therefore, non-invasive tools are mandatory to explore patients with AKI. In this setting, urinary protein profile can be helpful to differentiate tubular from glomerular injury. For these reasons, we conducted a retrospective monocenter study to assess the incidence and risk factors of AKI, and to describe the clinical and biological characteristics of these patients. For these patients, we performed specific urine protein assays investigating glomerular and tubular protein markers and assessed hemodynamic variables by echocardiography.

## 2. Materials and Methods

### 2.1. Setting

Our ICU is located in Henri Mondor hospital (Creteil, France), which is a 958 beds academic and tertiary referral hospital located in a 1.4 million citizens region (Val de Marne). At the time of the pandemic peak, our ICU was upgraded from one unit with 24 beds to four units with 80 beds. Patients without an immediate need for intubation were managed with continuous positive airway pressure outside the ICU in two intermediate care units.

Only one unit in the ICU was equipped for intermittent dialysis; patients hospitalized in the other units and who needed renal replacement therapy were managed with continuous veno-venous hemofiltration.

### 2.2. Patients

All consecutive adult patients (≥18 years), tested positive for SARS-CoV2 by polymerase chain reaction on a nasopharyngeal swab or a pulmonary sample, and hospitalized in our ICU from 3 March 2020 to 4 April 2020, were eligible. Patients with a history of end-stage kidney disease requiring chronic renal replacement therapy were excluded. This study was set in compliance with Helsinki Declaration and was approved by the ethical committee of the French Intensive Care Society. Owing to the observational nature of the study, patient’s consent was waived as per French law.

### 2.3. Renal Assessments

AKI was assessed during the entire ICU stay (or up to day-28 if the patient was still in ICU on day-28). AKI was defined according to the creatinine criteria of the Kidney Disease Improving Global Outcomes (KDIGO) [13]. Due to missing values of urine output, we assessed AKI in the main analysis using only creatinine values. However, we performed a sensitivity analysis involving the urine output (when available) in the definition of AKI. The estimated glomerular filtration rate (eGFR) before admission was calculated using the Chronic Kidney Disease Epidemiology (CKD-EPI) formula [14] and the known creatinine in the period from 6 months to 2 days before ICU admission, or if it was not known, using the ICU admission creatinine if it was normal. All creatinine baseline values could be collected for patients with abnormal creatinine at ICU admission. Urine protein profile included specific urine protein assays on a urine spot by immunonephelometry on IMMAGE-800^®^ analyzer (Beckman Coulter, Villepinte, France) [15,16], with the aim to investigate glomerular protein markers (such as albumin, transferrin, and immunoglobulin G), and tubular markers (such as alpha-1 microglobulin and retinol-binding protein). Proteins values are given as protein/urinary creatinine ratio (mg/mmol). Urine protein profile was performed for all patients when urine was available on day 1, 3, and 7 following ICU admission. Blood and urine samples were collected as part of routine care.

### 2.4. Echocardiography

In critically-ill patients, the hemodynamic status may impact kidney function via several mechanisms, including hypovolemia [17], hypoperfusion [18], and/or congestion [19,20,21,22,23]. In order to scrutinize these mechanisms, we performed hemodynamic phenotyping using bedside advanced critical care echocardiography, as previously proposed [24]. To evaluate cardiac function, transthoracic echocardiography (TTE) was performed within 72 h of ICU admission by trained operators (competent in advanced critical care echocardiography) using Vivid S70 and Vivid E9 systems (GEMS, Boston, MA, USA) and following a standard procedure [24]. The assessment of left (LV) and right ventricles (RV) size, contractility (by speckle tracking imaging), output, and loading conditions are detailed in the supplemental method (see Supplementary File S1). All measurements performed for each examination were averaged over a minimum of three cardiac cycles (five to ten in case of non-sinus rhythm).

### 2.5. Other Data Collected

We collected demographic (baseline comorbidities, usual treatments), clinical, and biological data, as well as COVID-19 course details. In addition, severity scores (Simplified Acute Physiologic Score II [SAPS II] and Sepsis-related Organ Failure Assessment [SOFA]), organ support measures (including mechanical ventilation, vasopressor support, extracorporeal membrane oxygenation), duration of ICU stay, and day-28 vital status were assessed.

### 2.6. Statistics

Descriptive statistics covered median and interquartile (IQR) for quantitative variables and numbers and percentages for categorical variables. We compared baseline patients’ characteristics and outcomes between AKI and no-AKI patients, and between deceased and alive patients using Chi-square or Fisher tests for categorical variables, and Mann–Whitney for quantitative variables.

To evaluate independent factors associated with AKI or 28-day mortality, significant univariate risk factors were examined using backward stepwise logistic regression analysis and Cox proportional hazards model with time-dependent covariates. To avoid overfitting, we considered that we could enter a maximal number of five variables in each model (to allow a minimum of 5 outcome events per predictor variable) [25]. Among related significant univariate factors, only the most statistically significant yet clinically relevant were entered into the regression models in order to minimize the effect of collinearity. The selection process was guided by maximal imbalances between groups, as estimated by absolute standardized differences, which are independent of the sample size and variable unit [26]. Coefficients were computed by the method of maximum likelihood. Selection of the model was performed using Lasso regression, with penalty factor determined by cross-validation and evaluation of the models by likelihood ratio tests. The evolution of urinary protein profile with time was performed with a Friedman test. All statistical tests were two-sided, and a *p*-value was considered statistically significant if <0.05. The statistical analyses were performed using JMP (v15.0.1), SPSS 24 (IBM Corp), and R 3.1.2 (The R Foundation for Statistical Computing, Vienna, Austria).

## 3. Results

### 3.1. Patients

During the study period, 71 patients with laboratory-confirmed COVID-19 were hospitalized in our ICU. One patient with end-stage kidney disease requiring chronic intermittent hemodialysis was excluded. The baseline characteristics at ICU admission of the 70 enrolled patients (56 men and 14 women) are summarized in Table 1. The median age was 60.5 (50–70.2). Body mass index was ≥30 kg/m² in 30 (42.8%) patients. The median time from symptoms onset to ICU admission was 8 (5–10) days.

### 3.2. AKI Prevalence and Risk Factors

Characteristics of patients with and without AKI are reported in Table 1. Overall, 45 (64.3%) patients developed AKI. The time from symptom onset and from ICU admission to AKI were 9 (6–14) and 2 (0–5) days, respectively. Only 6/45 (13%) patients developed an AKI after 7 days of ICU stay. There was no significant difference regarding clinical characteristics between groups, except for hypertension, which was more common (64.4% vs. 40%, *p* = 0.049), and pre-ICU eGFR, which was lower in patients with AKI than in those without (90 (72.5–101.5) vs. 104 (83.5–114.5), *p* = 0.03). At ICU admission, SAPSII score and shock presentation, expressed by the need for vasopressor, were higher in AKI patients (respectively 36 (28–45) vs. 27 (24–36), *p* = 0.005 and 57.8% vs. 20%, *p* = 0.002). The cycle threshold of SARS-CoV2 RT-PCR was similar in both groups. In multivariable analysis, the only factor associated with AKI was the need for vasopressor at ICU admission (OR = 5.47 (1.74–17.2), *p* < 0.01, Table S2 in Supplementary Materials).

### 3.3. Echocardiography

During the first 72 h of ICU admission, echocardiography was performed on 58 patients. Hemodynamic parameters were similar between AKI and no-AKI patients except for lower values of early-to-late (E/A) diastolic velocities ratio at mitral valve annulus in the AKI group (Table 2).

### 3.4. Urine Protein Profiles

**Figure 1:**
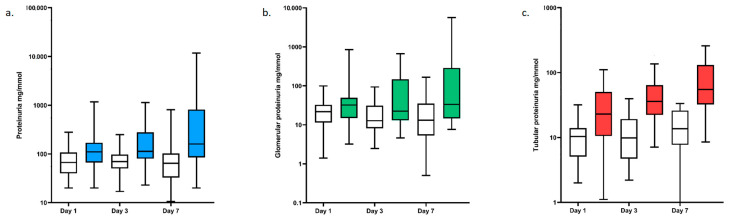
Boxplot of urine protein profile of COVID-19 critically ill patients on days 1, 3, and 7. (**a**) total proteinuria expressed as protein/creatinine ratios (median [IQR], min–max); white boxes: no-AKI patients; blue boxes: AKI patients; (**b**) glomerular proteinuria expressed as protein/creatinine ratios (median [IQR], min–max); white boxes: no-AKI patients; green boxes: AKI patients; (**c**) tubular proteinuria expressed as protein/creatinine ratios (median [IQR], min–max); white boxes: no-AKI patients; red boxes: AKI patients.

Urine protein profile was assessed on days 1, 3, and 7 of hospitalization for respectively 58, 48, and 45 patients (Table S1 in Supplementary Materials). Total and tubular proteinuria were higher on day 1 in AKI patients than in no-AKI patients, whereas glomerular proteinuria was similar in both groups (Figure 1). There was a significant increase with time in total and tubular proteinuria in patients with AKI (*p* values of 0.003 and 0.007, respectively), but not in those without AKI (*p*-values of 0.8 and 0.4, respectively). In contrast, the glomerular proteinuria did not change with time in both groups (*p*-values of 0.7 and 0.9, respectively) (Figure 1).

### 3.5. AKI Severity

AKI occurred after a median of 2 (0–5), 2 (0–4), and 0 (0–2) days of ICU admission, the start of mechanical ventilation, and presentation of shock, respectively. According to creatinine KDIGO criteria, AKI was classified as stage 1, 2, or 3 in four (8.9%), five (11.1%), and 36 (80%) patients, respectively. Overall, 23 (32.9%) patients needed renal replacement therapy, i.e., half of AKI patients. Renal replacement therapy was introduced after a mean of 9 ± 6 days of ICU admission. Ten patients (43.5%) were treated with intermittent hemodialysis and sixteen (69.5%) received continuous veno-venous hemofiltration; three patients had both therapies. The type of dialysis technique was imposed by the equipment available in the ICU sub-unit where a patient was hospitalized. The main reasons for dialysis initiation were hyperkalemia (*N* = 13, 56.5%), uremia (*N* = 4, 17.4%), anuria (*N* = 3, 13.1%), metabolic acidosis (*N* = 2, 8.7%), and fluid overload (*N* = 1, 4.3%). By 10 June, two AKI patients were still on dialysis.

### 3.6. Patient Outcomes

Patients’ general outcomes are reported in Table 3. No patient was lost to follow-up on day 28. Overall, AKI patients had a worse outcome than their counterparts with higher maximum doses of vasopressor, as they required more frequent mechanical ventilation, had a worse oxygenation, a lower survival during the ICU stay and on day 28, and prolonged ICU stay in survivors. Significant risk factors for 28-days mortality with logistic regression were AKI with the need for renal replacement therapy and non-renal SOFA score. The Cox regression with time-dependent covariates showed similar results, except for the association between congestive heart failure and death, which was marginally significant with the logistic regression and significant with the time-dependent Cox model (Table S2).

### 3.7. Sensitivity Analysis

A sensitivity analysis was performed using urine output (when available) in addition to creatinine criteria for the definition of AKI according to KDIGO. This analysis found that five patients classified as non-AKI with the sole creatinine criteria were classified as having AKI when considering urine output. This analysis found a higher prevalence of AKI (*N* = 50/70, 71.4%). However, the results were similar to the main analysis concerning factors associated with AKI (shock on day-1 and SAPS2) and factors associated with 28-days mortality (congestive heart failure, non-renal SOFA and AKI requiring renal replacement therapy) (Tables S3 and S4 in Supplementary Materials).

## 4. Discussion

In the present study, 64% of COVID-19 patients hospitalized in our ICU during the first wave of the pandemic developed an AKI and half of them required renal replacement therapy. Their urinary protein profile suggested a predominant tubular involvement. Shock at ICU admission was the main risk factor for AKI. AKI requiring renal replacement therapy was associated with 28-day mortality along with non-renal SOFA score and congestive heart failure.

The pathophysiological process underlying COVID-19-related AKI is still controversial. Contrary to previous studies [9], we were able to assess pre-ICU admission characteristics and to find an effect of comorbidities (hypertension and reduced pre-ICU eGFR) on the risk of developing AKI. These findings are consistent with reported results on AKI in other septic situations [27]. The main risk factor for AKI in our cohort was the presence of shock at ICU admission, suggesting that hemodynamic instability due to the tubular injury it provokes, might be a key marker to AKI development in severe COVID-19 [28]. The only echocardiographic parameter associated with AKI was a reduced E/A ratio in the mitral valve. This association may reflect the higher prevalence of hypertension in our AKI patients and/or the presence of hypovolemia, which could be one of the underlying mechanisms that trigger AKI in COVID-19 related sepsis. However, the interpretation of echocardiography data should be tempered down because of the high number of comparisons that may increase the risk of false-positive findings. Although AKI occurred rapidly after initiation of invasive mechanical ventilation (median of 2 days), mechanical ventilation parameters and pulmonary circulation dysfunction at ICU admission were not associated with AKI, suggesting no major role of a congestive mechanism in the first day of ICU hospitalization [29]. However, this result conflicts with others studies showing an association between ventilator parameters and AKI [30,31]. The monocentric design of our study may explain this discrepancy. More studies are required to evaluate the balance between the positive effects of PEEP on lung function and its potentially detrimental effects on renal function. Extensive analysis of urinary protein levels demonstrated higher tubular proteinuria in AKI patients on day 1 and a significant increase between day 1 and day 7 in this group. These data highly suggest that, in ICU patients, tubulointerstitial damage is the key parameter in the pathogenesis of COVID-19 associated AKI rather than glomerular injury. The development of shock coupled with hypoxemia at the initial phase of ICU admission enhances this hypothesis.

Urine protein analysis is a tool used to scrutinize pathophysiological processes of AKI in ICU where kidney biopsies are not easily feasible. Low molecular weight proteins (such as RBP) are freely filtered through the glomerular basal membrane. In a physiological situation, these proteins are almost completely (>99%) reabsorbed by proximal tubular epithelial cells, mainly by a molecular complex formed by megaline and cubilin [32]. Experimental sepsis reduces the expression of megalin in the proximal tubular cells [33]. In septic patients, a glomerular injury was also suggested, and albuminuria (detected either with urinary dipstick [34] or using the albumin/creatinine ratio [35,36]) seems to be a predictor of AKI and death. Our results of a prominent tubular proteinuria are consistent with those recently demonstrated by Kormann et al., who showed that 75% of COVID-19 patients had proximal tubular biological abnormalities [12], and with those of Rubin et al. who reported an albuminuria/proteinuria ratio <0.5 in patients with severe COVID-19 [11]. The underlying renal lesions of AKI in SARS-CoV-2 patients have been described in postmortem studies as typical features of acute tubular lesions affecting proximal tubules in a prominent and diffuse pattern, with vacuolar degeneration and loss of brush border [37,38]. COVID-19 associated glomerular lesions have been described in rare cases, showing collapsing glomerulopathy, a particularly aggressive form of glomerular injury particularly found in patients of African ancestry with genetic susceptibility risk factors [39]. Unfortunately, and owing to the compromised vital status and hemodynamic instability of our patients, renal biopsy was taken from only one patient who had significant proteinuria and AKI. The biopsy showed typical pathological lesions of severe glomerular endotheliopathy associated with acute tubular necrosis.

In this study, we classified AKI for the main analysis only using the creatinine criteria of the KDIGO guidelines and not the urine output criteria because of missing data. This strategy may have underestimated the rate of AKI. The sensitivity analysis involving available urine output data showed a higher prevalence of AKI. However, factors associated with AKI and 28 days mortality were similar in the main and sensitivity analyses.

The incidence of AKI observed in the current study is high compared to previous studies involving a mixed population with 10–16% critically-ill patients [7,8] but is in line with the rate of 40 to 80% observed in critically-ill COVID-19 [10,11,12,38]. This rate of AKI seems higher than previously reported in non-COVID-19 ARDS [40,41] and even higher than reported in the setting of septic [42] or cardiogenic shocks [43]. Even if shock and hemodynamic instability are probably the main factors for COVID-19 associated AKI in ICU, this discrepancy could suggest a specific role of SARS-CoV2 in the pathogenesis of kidney damage. Such a hypothesis is supported by the fact that severe kidney injury may occur in patients with moderate lung symptoms and may persist even after the pulmonary symptoms have resolved [39]. We found no association between the respiratory viral load of SARS-CoV2 and AKI. SARS-CoV2 receptors, angiotensin-converting enzyme2 (ACE2), and transmembrane serine 2 protease (TMPRSS2), which may play a key role in intracellular invasion, are constitutively expressed and possibly upregulated on the brush border of the proximal tubule and in podocytes of the kidneys of COVID-19 patients [44]. A retrospective postmortem study analyzing kidneys from patients who died from severe COVID-19 has reported the in situ expression of viral nucleocapsid protein (NP) antigen in the tubular compartment [37]. Consistently with a potential direct cytopathic effect of the virus, Puelles et al. detected SARS-Cov2 RNA and protein in glomerular epithelial and tubular cells extracted from autopsy samples [45]. In contrast, other reports failed to detect SARS-CoV-2 RNA by in situ hybridization in AKI patients with heavy proteinuria [38,46,47]. Of note, the use of potentially nephrotoxic drugs was not different between AKI and non-AKI patients in our study. Other mechanisms could be involved in AKI, such as systemic inflammatory response with inflammatory cells infiltration following the cytokines storm [48], complement activation leading to endothelial and tubular damage [49], micro-thrombus formation, and subsequent renal infarction [48,50]. Lastly, a transcriptomic study described a higher expression of the viral receptor ACE2 in the kidney cells of healthy European volunteers as compared to healthy Asian volunteers [44]. This difference could explain the higher AKI rate in our French cohort as compared to Chinese studies.

AKI patients presented a higher rate of mechanical ventilation, a higher dose of catecholamine, and a longer ICU stay for survivors. AKI with the need for dialysis was strongly associated with morbidity and mortality in our study as well as in other studies evaluating AKI in hospitalized COVID-19 patients [38]. These results are concordant with several studies in ICU showing that AKI is associated with increased morbidity.

The strength of our study lies in the detailed phenotyping of urine proteins and hemodynamic parameters. Nonetheless, our work has several limitations. First, it is monocentric with a limited sample size; further multicentric studies are needed to validate our findings. Second, renal biopsies could not be performed to better scrutinize the mechanisms of AKI. Third, a direct hemodynamic assessment of renal blood flow would have enriched the hemodynamic study. Last, urine protein profile and echocardiography measurements could not be assessed for all patients and we could not report the long-term prognosis of AKI. Over the past few years, AKI has emerged as a risk factor for chronic kidney disease in hospitalized and ICU patients [51] and it will be interesting to assess renal function in COVID-19 survivors in the long term.

## 5. Conclusions

In conclusion, AKI is a common and dreaded complication of severe COVID-19. Shock is a major risk factor and tubular involvement seems prominent, suggesting that in ICU, hemodynamic instability is probably the crucial factor responsible for AKI. Further studies are needed to test strategies aimed at preventing AKI in critically-ill COVID-19 patients.

## Figures and Tables

**Table 1:** Characteristics of critically-ill COVID-19 patients according to occurrence of acute kidney injury (AKI).

	All Patients (*N* = 70)	No AKI (*N* = 25)	AKI (*N* = 45)	*p* Value
Clinical characteristics				
Age (years), median (IQR)	60.5 (50–70.2)	58 (47.5–70)	61 (54.5–70.5)	0.25
Female sex	14 (20%)	3 (12%)	11 (24.5%)	0.35
Obesity	30 (42.8%)	9 (36%)	21 (46.7%)	0.45
Body mass index, kg/m^2^	29 ± 5.5	28.9 ± 4	29.1 ± 6.1	0.84
Hypertension	39 (55.7%)	10 (40%)	29 (64.4%)	**0.049**
Diabetes mellitus	25 (35.7%)	9 (36%)	16 (35.6%)	0.97
Congestive heart failure	8 (11.4%)	1 (4%)	7 (15.6%)	0.24
Chronic kidney disease	11 (15.7%)	4 (16%)	7 (15.6%)	0.96
Pre-ICU eGFR, mL/min/1.73 m^2^	93 (78.5–105.5)	104 (83.5–114.5)	90 (72.5–101.5)	**0.03**
Time from symptom onset to ICU admission, days	8 (5–10)	10 (6.5–11)	7 (4–10)	0.075
Time from symptom onset to AKI, days			9 (6–14)	
Time from ICU admission to AKI			2 (0–5)	
Treatment before ICU				
Steroid	3 (4.3%)	0 (0%)	3 (6.7%)	0.55
ACE inhibitor	13 (18%)	2 (8%)	11 (24.4%)	0.12
ARB	13 (18%)	5 (20%)	8 (17.8%)	>0.99
Diuretic	13 (18%)	5 (20%)	8 (17.8%)	0.82
Organ failures at ICU admission				
SAPSII	33 (27–43)	27 (24–36)	36 (28–45)	**0.005**
Non-renal SOFA score	4 (3–6)	5 (3–7)	7 (5–8)	**0.017**
Need for inotrope	5 (7.1%)	0 (0%)	5 (11.1%)	0.15
Need for vasopressor	31 (44.3%)	5 (20%)	26 (57.8%)	**0.002**
Fluid balance on day 1 (*N* = 64)	758 (205–1366)	505 (72–1200)	920 (220–1587)	0.24
Cycle threshold of SARS-COV-2	29.3 ±5.9	30.2 ± 4.7	28.9 ± 6.5	0.33
Ventilation at ICU admission				
Invasive mechanical ventilation	49 (70%)	15 (60%)	34 (75.6%)	0.17
Mean positive end expiratory pressure, cmH_2_ O	12 (9–12)	10 (9–12)	12 (9–12)	0.49
PaO_2_/FiO_2_ ratio, mmHg	120 (97–160)	123 (94–173)	120 (98–162)	0.95
Respiratory system compliance	32 (25–40)	33 (29–40)	30 (25–40)	0.66
Treatment in ICU (before AKI or within the 7 days after ICU admission)				
Aminoglycoside	6 (8.5%)	2 (8%)	4 (8.9%)	>0.99
Glycopeptide	1 (1.4%)	0 (0%)	1 (2.2%)	>0.99
Iodinated contrast agent	28 (40%)	13 (52%)	15 (33.3%)	0.13
SARS-CoV2 specific treatment				0.43
None	10 (14.3%)	2 (8%)	8 (17.8%)	
Lopinavir-Ritonavir *	26 (37.1%)	8 (32%)	18 (40%)	
Hydroxychloroquine *	28 (40%)	12 (48%)	16 (35.6%)	
Tocilizumab *	5 (7.1%)	3 (12%)	2 (4.4%)	
Remdesivir	1 (1.4%)	0	1 (2.2%)	

* alone or in combination; pre-ICU eGFR, estimated glomerular filtration rate before intensive care unit admission, estimated according to Chronic Kidney Disease Epidemiology Collaboration (CKD-EPI) formula; ICU, intensive care unit; SAPSII, Simplified Acute Physiologic Score II; SOFA, Sepsis-related Organ Failure Assessment score; PaO_2_/FiO_2_ ratio, ratio of partial oxygen pressure in arterial blood to fraction of inspired oxygen; ACE, angiotensin converting enzyme; ARB, angiotensin II receptor blocker. Significant *p*-value are shown in bold characters.

**Table 2:** Echocardiographic characteristics of critically-ill COVID-19 patients according to the occurrence of acute kidney injury (AKI).

	All Patients (*N* = 58)	No AKI (*N* = 17)	AKI (*N* = 41)	*p* Value
Preload				
Inferior vena cava maximal diameter (mm)	22 (18–25)	22 (15–25)	23 (20–26)	0.24
E/A ratio at mitral valve	1 (0.78–1.3)	1.2 (0.8–1.5)	0.9 (0.7–1.1)	**0.02**
E/e’ ratio at lateral mitral annulus	7 (5.9–8.6)	6.5 (4.9–7.8)	7.4 (6.3–9.9)	0.12
Contractility				
LVEF (%)	60 (49–57)	62 (54–70)	55 (42–63.5)	0.06
Global LV longitudinal peak systolic strain (%)	−14.8 (−17.9 to −10.2)	−15.8 (−18.2 to −11.2)	−11.9 (−18.1 to −9.1)	0.31
Tissue Doppler peak systolic wave at mitral lateral annulus (cm. s^−1^)	11 (9–13.3)	11 (9–14.5)	11 (8.3–12.8)	0.75
Ventricular-arterial coupling	1.9 (1–3)	2 (1.5–3.6)	1.9 (1–3)	0.24
LV end-systolic maximal elastance (mmHg.mL^−1^)	3.6 (1.8–5)	4.2 (2.8–5.5)	3.6 (1.8–4.8)	0.33
Afterload				
End-systolic arterial elastance (mmHg.mL^−1^)	1.75 (1.59–2.12)	1.7 (1.58–1.8)	1.8 (1.6–2.2)	0.16
Systemic vascular resistance (mmHg.L^−1^.min)	1096 (907.5–1245)	1077 (919–1289)	1096 (900–1248)	0.7
RV function				
Tissue annular plane systolic excursion (mm)	21 (18–25)	22 (19.5–25)	21 (18.5–25)	0.3
Tricuspid systolic wave (cm/s)	13 (11–17)	13 (11.5–16)	13.5 (11–17)	0.8
Pulmonary vascular dysfunction, n (%)				
Absent	25 (43.1%)	7 (41.2%)	18 (43.1%)	0.95
Moderate	4 (6.9%)	1 (5.9%)	3 (7.3%)	
Severe (cor pulmonale),	29 (50%)	9 (52.9%)	20 (48.8%)	
Global function				
Cardiac index (mL min^−1^ m^−2^)	2.9 (2.4–3.5)	2.9 (2.3–3.4)	3 (2.4–3.7)	0.63

E/A, early to late diastolic velocities ratio; LVEF, left ventricle ejection fraction; LV, left ventricle; E/e’, ratio of early mitral inflow velocity to mitral annular early diastolic velocity. Significant *p*-value are shown in bold characters.

**Table 3:** Outcomes of critically-ill COVID-19 patients according to the occurrence of acute kidney injury (AKI).

	All Patients (*N* = 70)	No AKI (*N* = 25)	AKI (*N* = 45)	*p* Value
Circulation				
Need for dobutamine during ICU stay *	10 (14%)	0 (0%)	5 (11.1%)	0.15
Need for vasopressor during ICU stay *	43 (61%)	7 (28%)	6 (13.3%)	0.19
Maximal dose of norepinephrine, mg/h	3 (1.5–6.3)	1.4 (1–3.5)	7.5 (3.8–9.8)	**0.013**
Ventilation				
Invasive mechanical ventilation	61 (87%)	18 (72%)	43 (95.6%)	**0.008**
PaO_2_/FiO_2_ ratio, mmHg	85 (68–104)	98 (82–126)	75 (65–98)	**0.008**
Mean positive end expiratory pressure, mmHg	10.7 ± 2	9.1 ± 1.7	11.1 ± 2	**0.014**
Extra corporeal membrane oxygenation	17 (24.3%)	3 (12%)	14 (31.2%)	0.088
General outcomes				
Duration of ICU stay in survivors on day 28, days	20 (7–34)	12 (6–18)	34 (25–49)	**<0.0001**
Death in ICU	25 (35.7%)	1 (4%)	24 (53.3%)	**<0.0001**
In-hospital death	26 (37.1%)	1 (4%)	25 (55.6%)	**<0.0001**
Alive on day 28	46 (65.7%)	24 (96%)	22 (48.9%)	**<0.0001**
Still in ICU on day 28 (n = 46)	16/46 (34%)	3/24 (12.5%)	13/22 (59.1%)	**<0.001**

* Excluding patients fulfilling the condition before the episode of acute kidney injury; PaO_2_/FiO_2_ ratio, ratio of partial oxygen pressure in arterial blood to fraction of inspired oxygen; ICU, intensive care unit. Significant *p*-values are shown in bold characters.

## Data Availability

The datasets used and/or analyzed during the current study are available from the corresponding author on reasonable request. The datasets supporting the conclusions are included within the article.

## Supplementary Materials

The following are available online at https://www.mdpi.com/article/10.3390/jcm11072029/s1, Supplementary file S1: Supplemental method of echocardiography procedure. Supplementary file S2: Table S1: Urinary protein profile on day 1 of critically-ill COVID-19 patients, with or without acute kidney injury (AKI). Table S2: Factors associated with acute kidney injury (AKI) and with death at 28 days in critically-ill COVID-19 patients. Table S3: Univariate and multivariable analysis of factors associated with acute kidney injury (AKI, defined taking into account available urine output data), Table S4: Univariable and multivariable analysis of factors associated with 28 days mortality. References [52,53,54,55,56,57,58,59,60,61,62,63] are cited in the supplementary materials.

## Abbreviations

ACE inhibitor	angiotensin-converting enzyme inhibitor
ACE2	angiotensin-converting enzyme 2
ARB	angiotensin II blocker
ARDS	acute respiratory distress syndrome
BMI	body mass index
COVID-19	coronavirus disease-19
CVVHF	continuous veno-venous hemofiltration
ICU	intensive care unit
IgG	immunoglobulin G
IHD	intermittent dialysis
IQR	interquartile
KDIGO	kidney disease: improving global outcomes
PEEP	positive end expiratory pressure
RBP	retinol-binding protein
RT-PCR	reverse transcription-polymerase chain reaction
SAPS2	simplified acute physiology score 2
SARS-CoV2	severe acute respiratory syndrome-coronavirus 2
SD	standard deviation
SOFA	Sepsis-related Organ Failure Assessment score
TMPRSS2	transmembrane serine 2 protease
