# Supplementary material for: Acute Kidney Injury in Critically-Ill COVID-19 Patients
Source: J Clin Med. 2022 Apr 5;11(7):2029. doi: 10.3390/jcm11072029 (PMC8999255; doi:10.3390/jcm11072029)
Supplement: Supplementary file 1 [file jcm-11-02029-s001.zip › Supplementary File 1.pdf]

Acute kidney injury in intensive care COVID-19 patients: a monocenter retrospective study

Romain ARRESTIER[1,2], Ségolène GENDREAU[1,2], David MOKRANI[1], Jean-Philippe BASTARD[3,4,5], Soraya FELLAHI[4,5], François BAGATE[1,2], Paul MASI[1,2], Thomas d'HUMIERES[6], Keyvan RAZAZI[1,2], Guillaume CARTEAUX[1,2,7], Nicolas DE PROST[1,2], Vincent AUDARD[7,8*], Armand MEKONTSO DESSAP[1,2,7*]

Electronic supplement

PATIENTS AND METHODS

Echocardiography

To evaluate cardiac function, we used transthoracic echocardiography performed by trained operators (competent in advanced critical care echocardiography) [1] using Vivid S70 and Vivid E9 systems(GEMS, Boston, MA)with a standard procedure [2]. Briefly, the following echocardiographic views were examined: four-chamber and two-chamber long-axis views to assess left ventricle ejection fraction (LVEF, computed from LV volume using bi-plane Simpson method [1] when image quality was good, or

visually estimated when image quality was not sufficient to identify the endocardium) [2], tissue Doppler peak systolic wave at the lateral valve annulus of mitral and tricuspid valves[3], right ventricle size (a dilated RV was defined by a RV/LV end-diastolic area ratio ≥0.6)[4], long-axis M-mode view of the inferior vena cava to assess its maximal diameter [5], LV filling pressures [using pulsed-wave Doppler early (E) and late (A) diastolic wave velocities at the mitral valve, and tissue Doppler early (e') diastolic wave velocity at the lateral mitral valve annulus][6]. Pulsed-wave Doppler flows were obtained at the aortic valve to assess aortic velocity-time integral for cardiac output computation.Pulmonary circulatory system dysfunction was classified absent when pulmonary artery systolic pressure was <40 mmHg with normal right ventricle size and normal septal kinetics; moderate if pulmonary artery systolic pressure was ≥40 mmHg, orin case of dilated right ventricle,or septal dyskinesia without cor pulmonale; or severe in case of cor pulmonale (association of dilated right ventricle or septal dyskinesia) [7]. Echocardiographic images were digitally stored, and a computer-assisted evaluation was performed off-line by two trained operators (FB, PM). Echocardiography was performed within 72 hours of intensive care unit admission. All measurements were averaged over a minimum of three cardiac cycles (five to ten in case of non-sinus rhythm).Apical long-axis (four- and two-chamber) clips underwent on-line speckle tracking analyses by two trained operators (FB, PM). The average of three consecutive heart cycles was used to calculate global longitudinal peak systolic strain of the LV.

Assessment of contractility and loading conditions

Preload was assessed using estimates of LV filling pressures (E/A and E/e' ratios) [6]and maximum diameter of inferior vena cava [7]. Afterload was assessed using the following indices: ii) systemic vascular resistance (the most commonly used

measurement of vascular tone)= $\frac{80 * meanarterialpressure\ (mmHg)}{cardiacoutput (L.min^{-1})}$[8];and ii) end-systolic arterial elastance (to reflect the pulsatile component of peripheral load)= $\frac{systolicarterialpressure\ (mmHg)}{strokevolume\ (mL)}$[9–11].

LV systolic function was assessed using indices obtained by two-dimensional echocardiography (to calculate LVEF), tissue Doppler imaging (tissue Doppler peak systolic wave at the lateral mitral valve annulus), speckle tracking imaging (global longitudinal peak systolic strain of the LV), and two additional indices to reflect net cardiovascular performance and heart-arterial interaction, respectively, as:

i) LV end-systolic maximal elastance $= \frac{systolicarterialpressure\ (mmHg)}{LVend-systolicvolume\ (mL)}$[12,13], and ii) ventricular-arterial coupling, which is the ratio of LV end-systolic maximal elastance and end-systolic arterial elastance, namely $= \frac{stroke\ volume (mL)}{LVend-systolic\ volume\ (mL)}$[13]. LV end-systolic volume was obtained from the Simpson method [3] and stroke volume was derived from left ventricle outflow track diameter and aortic velocity-time integral.

REFERENCES

1. Lang RM, Bierig M, Devereux RB, Flachskampf FA, Foster E, Pellikka PA, et al. Recommendations for chamber quantification: a report from the American Society of Echocardiography's Guidelines and Standards Committee and the Chamber Quantification Writing Group, developed in conjunction with the European Association of Echocardiography, a branch of the European Society of Cardiology. J Am Soc Echocardiogr Off Publ Am Soc Echocardiogr. 2005;18:1440–63.

2. Gudmundsson P, Rydberg E, Winter R, Willenheimer R. Visually estimated left ventricular ejection fraction by echocardiography is closely correlated with formal quantitative methods. Int J Cardiol. 2005;101:209–12.

3. Seo J-S, Kim D-H, Kim W-J, Song J-M, Kang D-H, Song J-K. Peak systolic velocity of mitral annular longitudinal movement measured by pulsed tissue Doppler imaging as an index of global left ventricular contractility. Am J Physiol Heart Circ Physiol. 2010;298:H1608-1615.

4. Boissier F, Katsahian S, Razazi K, Thille AW, Roche-Campo F, Leon R, et al. Prevalence and prognosis of cor pulmonale during protective ventilation for acute respiratory distress syndrome. Intensive Care Med. 2013;39:1725–33.

5. Vieillard-Baron A, Evrard B, Repessé X, Maizel J, Jacob C, Goudelin M, et al. Limited value of end-expiratory inferior vena cava diameter to predict fluid responsiveness impact of intra-abdominal pressure. Intensive Care Med [Internet]. 2018 [cited 2018 Jan 26]; Available from: http://link.springer.com/10.1007/s00134-018-5067-2

6. Nagueh SF. Non-invasive assessment of left ventricular filling pressure: Non-invasive estimation of LV filling pressure. Eur J Heart Fail. 2018;20:38–48.

7. Boissier F, Katsahian S, Razazi K, Thille A, Roche-Campo F, Leon R, et al. Prevalence and prognosis of cor pulmonale during protective ventilation for acute respiratory distress syndrome. Intensive Care Med. 2013;39:1725–33.

8. Greim CA, Roewer N, Schulte am Esch J. Assessment of changes in left ventricular wall stress from the end-systolic pressure-area product. Br J Anaesth. 1995;75:583–7.

9. Sunagawa K, Maughan WL, Burkhoff D, Sagawa K. Left ventricular interaction with arterial load studied in isolated canine ventricle. Am J Physiol. 1983;245:H773-780.

10. Devereux RB. Toward a more complete understanding of left ventricular afterload. J Am Coll Cardiol. 1991;17:122–4.

11. Sandler H, Dodge HT. LEFT VENTRICULAR TENSION AND STRESS IN MAN. Circ Res. 1963;13:91–104.

12. Bombardini T, Costantino MF, Sicari R, Ciampi Q, Pratali L, Picano E. End-systolic elastance and ventricular-arterial coupling reserve predict cardiac events in patients with negative stress echocardiography. BioMed Res Int. 2013;2013:235194.

13. Sagawa K, Suga H, Shoukas AA, Bakalar KM. End-systolic pressure/volume ratio: a new index of ventricular contractility. Am J Cardiol. 1977;40:748–53.
