# Supplementary material for: Acute Kidney Injury in Critically-Ill COVID-19 Patients
Source: J Clin Med. 2022 Apr 5;11(7):2029. doi: 10.3390/jcm11072029 (PMC8999255; doi:10.3390/jcm11072029)
Supplement: Supplementary file 1 [file jcm-11-02029-s001.zip › Supplementary File 2.pdf]

**Table S1. Urinary protein profile of critically-ill COVID-19 patients, with or without acute kidney injury (AKI, defined taking into account available urine output data), median [IQR]**				
	**Total**	**No AKI**	**AKI**	**p**
**Day 1**	**N=58**	**N=20**	**N=38**	
Proteinuria (mg/mmol)	95.5 [60-131.5]	66.7 [40-107.5]	110 [66.75-170]	**0.013**
Albuminuria(mg/mmol) *(normal value < 3)*	20.9 [10-49]	15.1 [9.8-36.6]	32.1 [10.2-52.1]	0.14
Transferrin (mg/mmol) *(normal value < 0.2)*	1.1 [0.2-2.2]	1.1 [0.4-2.1]	1.1 [0.1-2.3]	0.85
Immunoglobulin G (mg/mmol) *(normal value < 1.13)*	2.8 [1.1-5.9]	1.8 [0.88-4.8]	3.7 [1.2-6.7]	0.10
Glomerular proteinuria (mg/mmol)	23.2 [12.4-46]	21.7 [11.4-32.6]	32.3 [15-49.6]	0.20
Alpha 1 microglobulin (mg/mmol) *(normal value < 2.23)*	12 [6.4-24.6]	9.8 [5-12.1]	17.1 [8.9-62.3]	**0.006**
Retinol binding protein (mg/mmol) *(normal value < 0.08)*	1.7 [0.3-4.6]	0.34 [0.08-3.5]	3.4 [1.2-13]	**0.0005**
Tubular protein (mg/mmol)	14 [7.1-31]	10.4 [5-13.9]	22.9 [10.6-50.3]	**0.002**
Albumin / protein ratio (%)	28.9 [13-36]	26.9 [13.535.5]	28.9 [11.5-39.4]	0.72

Table S2. Factors associated with acute kidney injury (AKI,defined taking into account available urine output data), and with death at 28 days in critically-ill COVID-19 patients

Variable	Absolute standardised difference (%)	Univariable logistic regression	Multivariable logistic regression	Multivariable Cox regression with time-dependent covariates
ACUTE KIDNEY INJURY				
Hypertension	50.4	2.72 [0.99-7.44], p=0.05	I/NR	I/NR
Pre-ICU eGFR, mL/min/1.73m²	48.1	0.98 [0.96-1.00], p=0.06	I/NR	I/NR
SAPSII	74	1.08 [1.02-1.15], p<0.01	I/NR	1.03 [1.01- 1.06], p=0.008
Need for vasopressor at ICU admission	84.1	5.47 [1.74-17.2], p<0.01	5.47 [1.74-17.2], p<0.01	2.67 [1.38-5.18], p=0.003
28-DAY MORTALITY				
Cardiovascular comorbidities				
Hypertension	78.8	4.94 [1.57-15.52], p=0.006	NI	NI
Diabetesmellitus	74.6	4.46 [1.55-12.82], p=0.006	NI	NI
Congestive heartfailure	79.9	18.53 [2.12-162], p=0.008	I/NS	3.61 [1.17-11.13], p=0.03
ACE inhibitor	72.7	6.3 [1.69-23.5], p=0.006	I/NS	I/NS
Diuretic	56.1	4.1 [1.16-14.42], p=0.028	NI	NI
Severity score at ICU admission				
SAPSII	111.5	1.131 [1.06-1.21], p<0.01	NI	NI
Age, years	83.1	1.076 [1.02-1.13], p=0.04	NI	NI
Non-renal SOFA score	125.5	1.85 [1.36-2.52], p<0.01	1.91[1.29-3.11], p<0.01	1.71[1.34-2.19], p<0.001
Fluid balance at day 1	57.9	1.001 [1-1.001] p=0.03	NI	NI
Hemodynamic support				
Need for inotrope at ICU admission	51.2	9 [0.95-85.7], p=0.056	NI	NI
Need for inotrope during ICU stay	62.1	5.9 [1.37-25.53], p=0.018	NI	NI
Need for vasopressor at ICU admission	88.3	5.55 [1.89-16.37], p=0.002	NI	NI
Need for vasopressor during ICU stay	93.7	7.64 [2-29.2], p=0.03	I/NR	I/NR
Respiratory support				
Invasive mechanical ventilation at ICU admission	63.8	4.5 [1.17-17.3], p=0.003	NI	NI
Kidneyfunction				
Pre-ICU eGFR, mL/min/1.73m²	59.4	0.98 [0.95-0.99], p=0.023	NI	NI
Acute kidneyinjury	126.1	25.1 [3.12-201.6], p=0.002	NI	NI
Acute kidney injury with KDIGO 3 stage	86.9	12.57 [1.41-111.7], p=0.023	NI	NI
Acute kidney injury with need for renal replacement therapy *	156.7	18 [4.99-64.9], p<0.001	24.2 [5.04-177.48], p<0.001	1.58[1.06-2.37], p= 0.03

[26]Selection of variables entered into the model was based on maximal imbalances between groups; NI, not included in the model; I/NR, included, but not retained in the final model; I/NS, included, but not significant in the final model.* Time-dependent covariate.

Table S3. Univariable and multivariable analysis of factors associated with acute kidney injury (AKI, defined taking into account available urine output data)

Variable	Univariable analysis HR [95%IC]	Multivariable analysis* HR [95%IC]
ACUTE KIDNEY INJURY		
Hypertension	2.04 [1.14-3.66], p=0.016	1.7 [0.84-3.4], p=0.1
Pre-ICU eGFR, mL/min/1.73m²	0.985 [0.972-0.999], p=0.032	1 [0.98-1], p=0.9
SAPSII	1.05 [1.03-1.07], p<0.00001	**1.07 [1.02-1.1], p=0.0009**
Need for vasopressor at ICU admission	3.65 [2.01-6.62], p=0.0002	**2.5 [1.32-4.6], p=0.005**

*Fine and Gray model with death as a competing risk

Table S4. Univariable and multivariable analysis of factors associated with 28 days mortality (AKI, defined taking into account available urine output data)

Variable	Univariable analysis HR [95%IC]	Multivariable analysis* HR [95%IC]
28-DAY MORTALITY		
Cardiovascular comorbidities		
Hypertension	3.96 [1.47-10.6], p=0.006	NI
Diabetesmellitus	3.36 [1.49-7.58], p=0.003	NI
Congestive heartfailure	7.81 [3.08-19.8], p=<0.0001	5.43 [1.17-11.13], p=0.002
Diuretic	3.31 [1.41-7.78], p=0.006	NI
Severity score at ICU admission		
SAPSII	1.08 [1.05-1.11]? P<0.0001	NI
Age, years	1.06 [1.02-1.1], p=0.004	NI
Non-renal SOFA score	1.63 [1.34-2], p<0.0001	1.74 [1.34-2.19], p<0.001
Hemodynamic support		
Need for inotrope during ICU stay	3.82 [1.57-9.27], p=0.003	NI
Need for vasopressor at ICU admission	4.42 [1.83-10.7], p=0.0009	NI
Respiratory support		
Invasive mechanical ventilation at ICU admission	3.79 [1.13612.7], p=0.03	NI
Kidneyfunction		
Pre-ICU eGFR, mL/min/1.73m²	0.98 [0.97-0.99], p=0.006	NI
Acute kidney injury with need for renal replacement therapy *	9.32 [3.08-19.8], p<0.001	1.69 [1.06-2.37], p= 0.0006

*Cox regression with time-dependent covariates
